# Cumulative oxygen consumption during development of two postharvest insect pests: *Callosobruchus maculatus* Fabricius and *Plodia interpunctella* Hübner

**DOI:** 10.1016/j.jspr.2018.03.006

**Published:** 2018-06

**Authors:** Hannah E. Quellhorst, Scott B. Williams, Larry L. Murdock, Dieudonne Baributsa

**Affiliations:** a Department of Entomology, Purdue University, West Lafayette, IN, United States; b Spensa Technologies, INC., West Lafayette, IN, United States

**Keywords:** Oxygen consumption, Insect development, *Callosobruchus maculatus*, *Plodia interpunctella*, Postharvest

## Abstract

Insect pests such as *Callosobruchus maculatus* Fabricius and *Plodia interpunctella* Hübner cause substantial losses to grain during postharvest storage. In the last few years, hermetic storage technologies have been successfully used by smallholder farmers in Africa and Asia to protect their harvested grain against insect pests. Hermetic technologies owe much of their effectiveness to restricting oxygen availability to insects confined in the containers. There is a need to better understand the biology of specific storage insect pests and their responses to hypoxia. We employed a novel and non-invasive analytical technology, the OxySense 5250i, to measure oxygen levels in closed containers, and evaluated its effectiveness in measuring the total oxygen consumption of two insect pests during their development: *C. maculatus* and *P. interpunctella*. The total amount of oxygen consumed by *C. maculatus* during its larval development period determined with the OxySense apparatus was not different from that previously recorded using another instrument, the Mocon Pac Check 325 gas analyzer. Using the OxySense 5250i, we found that *P. interpunctella* consumes nearly three times as much oxygen per insect over its larval-to-adult developmental period compared to *C. maculatus*. Information on the lifetime oxygen consumption of insects provides relevant information to the effectiveness and ability of hermetic technologies to protect stored products against insect pests.

## Highlights

• The OxySense 5250i is an effective method for measuring insect's oxygen consumption.
• On average, *Callosobruchus maculatus* consumes 8.3 ml of oxygen during its lifetime.
• *Plodia interpunctella* consumes about 3 times (26.9 ml) the oxygen of *C. maculatus*.
• Both *P. interpunctella* and *C. maculatus* have similar average daily oxygen consumption rates.

## 1 Introduction

Cereal grains and legumes comprise the majority of the staple subsistence crops of African farmers, accounting for 60–80% of the caloric intake (Awika, 2011). In West Africa, these staple food crops are produced by smallholder farmers who intercrop cereals with legumes such as cowpea with sorghum or millet (Singh et al., 1997). Despite efforts to increase production, these smallholder farmers do not reap the full benefits of their harvested crops due to losses during storage after harvest. Major postharvest insect pests such as *Callosobruchus maculatus* Fabricius and *Plodia interpunctella* Huebner damage stored food crops by feeding on the grain. This not only reduces the edible mass, but also damages the endosperm or seed germ, resulting in a loss of grain quality and seed viability (Malek and Parveen, 1989; Santos et al., 1990). Insect pests also contribute to secondary bacterial and fungal infestations (Sallam, 1999).

Hermetic technologies are viable and economical solutions to reduce postharvest storage losses and improve food security. Hermetic technologies arrest gas and moisture exchange between the internal and external environments. Living organisms that infest the grain, such as insects and fungi, deplete the available oxygen and cause an increase in carbon dioxide levels within the container (Bern et al., 2013). Low oxygen levels rather than high carbon dioxide levels contribute to insect mortality (Bailey, 1965; Navarro et al., 1994). Insects living in low oxygen environments depend on oxidative metabolism to generate the water they need for growth and development (Murdock et al., 2012). Without oxygen, their main water supply is blocked and their growth, development, and survival are arrested. In combination with reduced oxygen availability, lower moisture content of the grain leads to an increase in the mortality of the insects since the supply of water in this environment is very restricted (Navarro et al., 1994).

Oxygen consumption by insects can provide insight into the effectiveness and ability of hermetic technologies to protect stored products against major pests. The work of Murdock et al. (2012), measured the cumulative lifetime oxygen consumption of *C. maculatus* using a Mocon PAC Check^®^ Model 325 Headspace analyzer. *C. maculatus* utilized approximately 8.9 ± 0.4 ml of oxygen per insect from egg to emerging adult. It is important to understand how much oxygen additional stored products insect pests need in order to complete their developmental cycle. The Mocon PAC Check^®^ Model 325 Headspace analyzer measurements used by Murdock et al. (2012) involve puncturing an airtight container and removing a small air sample; this might be a possible source of error, especially when multiple readings are taken. Thanks to advances in technology, a non-invasive oxygen analyzer called the OxySense 5250i (OxySense, Las Vegas, NV, USA) has come into use for measuring the oxygen content in bottles, packages, and sealed containers.

Our objectives were to1: assess the effectiveness the OxySense 5350i, a new and non-invasive technology, in measuring oxygen consumption; and 2: investigate the oxygen requirements of *P. interpunctella* from egg to adult emergence.

## 2 Materials and methods

All experiments were carried out in the Purdue Improved Crop Storage (PICS) Laboratory at Purdue University (West Lafayette, IN, USA) in April and June of 2016, and April of 2017. The experiments involving *C. maculatus* took 23 days to complete and the *P. interpunctella* experiments took 45 days. *C. maculatus* were obtained from a colony maintained in a walk-in chamber at 25° C and 40% relative humidity (R.H.). *P. interpunctella* were obtained from the Stored Products Integrated Pest Management (IPM) laboratory at Purdue University. *P. interpunctella* were used to start new colonies in the PICS laboratory and were maintained on a cracked wheat diet in the same walk-in chamber as described above.

The OxySense 5250i was used to assess oxygen consumption by individual insects. The OxySense 5250i technology relies on a light sensitive oxygen sensor called the O_2_xyDot^®^. The OxyDot - O_2_xyDot^®^ contains a pigment that fluoresces under ultraviolet light. Oxygen quenches the fluorescence in proportion to its concentration. When less oxygen is present the OxyDot - O_2_xyDot^®^ fluoresces more intensely. The OxySense 5250i reads and interprets this fluorescence and displays a percentage value, which represents the oxygen level in a given volume. The oxygen sensors (OxyDots) are first attached to the inside of the container prior to sealing and then external measurements are made using the fiber-optic reader pen attached to the OxySense 5250i. Readings are taken by holding the fiber-optic reader pen over the OxyDot.

Measuring cumulative oxygen consumption from the egg stage to when the adult insect emerges requires obtaining fresh eggs of known age. Approximately 200 unsexed *C. maculatus* adults were removed from colony jars and allowed to oviposit on 200 cowpea seeds held in a glass Ball^®^ 16 ounce jar. *C. maculatus* adults were removed after 2 h. Seeds with two or more eggs initially had excess eggs scraped off using needlepoint tip forceps. The infested grain was held in isolation for five days and left undisturbed while egg development occurred. Female *C. maculatus* oviposit onto the surface of a seed leaving a translucent, elliptical egg. Five days post oviposition, the embryo becomes sufficiently large enough that a black spot (black head capsule stage) is visible. At this stage, it is possible to determine which eggs are viable. We selected a random sample of the cowpea seeds and examined them under a microscope to identify the presence of black head capsules. Forty cowpea seeds were selected that had one egg per seed. Each of the forty seeds were then placed into separate glass bottles (500 ml) pre-equipped with an O_2_xyDot^®^ sensor on their inside surfaces and labeled accordingly. The bottles were then sealed with a screw cap and the cap-bottle interface wrapped with Parafilm™ to ensure an airtight seal.

To obtain fresh *P. interpunctella* eggs, 15 unsexed adult moths were removed from colony jars and allowed to mate and oviposit in a breeding chamber. The breeding chamber consisted of an inverted glass Ball^®^ 16 ounce jar with a square of corrugated cardboard placed inside. A mesh screened lid was fitted onto the jar, along with a plastic deli cup container, which was fitted around the jar lid. The breeding chamber was inverted with the deli cup at the base. Female *P. interpunctella* oviposit eggs onto the surface of grain, but do not attach them to any surface. In this arrangement, once oviposited, the eggs fall down through the mesh screen and into the bottom of the deli cup. The larvae begin hatching in two to fourteen days (Rutschky and Calvin, 1990). Due to the variability in larval hatching time, the plastic container was removed from the breeding chamber and eggs were selected and examined under a microscope on day one post-oviposition. Only eggs that were single, not adhering to other eggs, and had a firm and healthy appearance were used. Once the one-day-old *P. interpunctella* eggs were collected and put in glass bottles (500 ml with 30 ml of cracked wheat) pre-equipped on their inner surfaces with an O_2_xyDot^®^ sensor. Due to their small size, forty *P. interpunctella* eggs were selected under a microscope and placed each in a 500 ml-bottle using a size 8 paintbrush. As with the *C. maculatus* bottles, the bottles were closed with a screw cap and wrapped with Parafilm™ to ensure an airtight seal.

The OxySense 5250i instrument was used to determine the initial percentage of oxygen present within each of the bottles. Readings were then taken every day until *C. maculatus* and *P. interpunctella* emerged as adults. The cumulative oxygen consumption (total amount of oxygen consumed) in milliliters (ml) per insect was calculated with the following formula: ((initial O_2_ percentage – final O_2_ percentage)/100) x volume of the bottle. We then calculated the average cumulative oxygen consumption (ml/insect) for both *C. maculatus* and *P. interpunctella*. The average cumulative oxygen consumption was calculated by adding up the cumulative oxygen consumption for each individual insect (replicate) and then dividing by the total number of insects (n = 22 for *C. maculatus*, n = 36 for *P. interpunctella*). We also calculated the maximum rate of oxygen consumption (ml/day/per insect), which is represented by the linear section with the steepest slope of the sigmoidal or logistic model. Finally, we calculated the average daily rate of oxygen consumption (ml/day/per insect) by dividing the average cumulative oxygen consumption by the average number of days it took for all insects to complete development cycle (from egg to adult emergence). A Mann-Whitney *U* test was used to compare the average cumulative oxygen consumption of *C. maculatus* with Murdock et al. (2012)’s original finding. A Mann-Whitney *U* test was also used to compare the average daily rate of oxygen consumption, the maximum rate of oxygen consumption, and the average cumulative oxygen consumption between the two insect species (*C. maculatus* and *P. interpunctella*). The graph was created using Sigma Plot 13 software (SYSTAT Software, Inc.; Point Richmond, CA).

## 3 Results

**Fig. 1:**
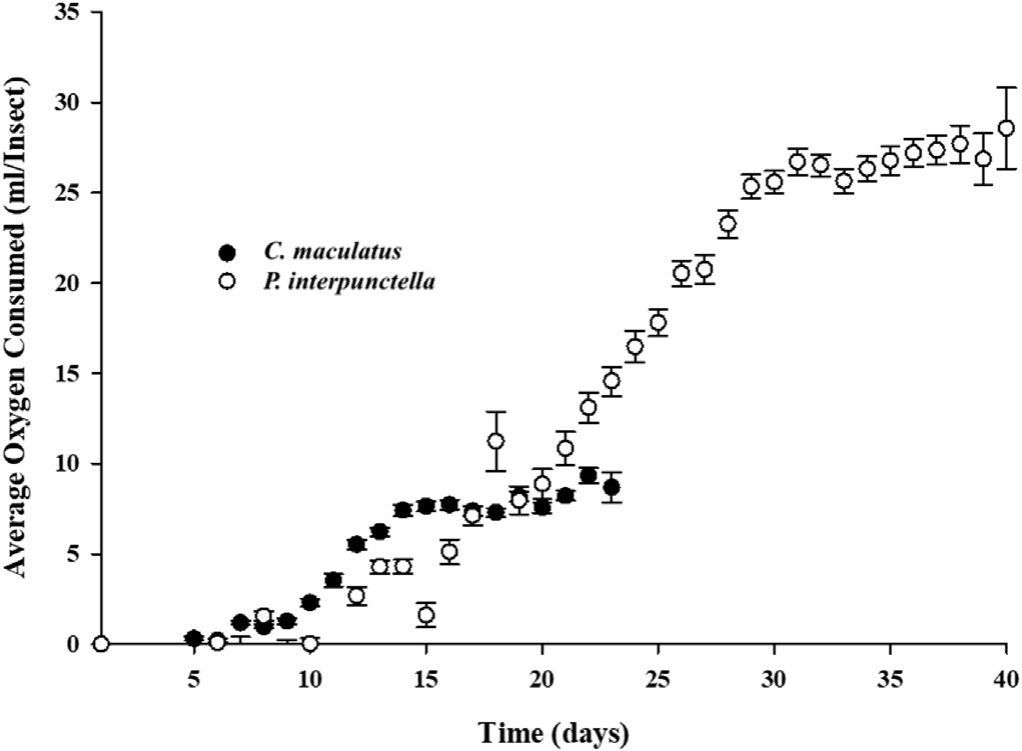
Average oxygen consumption of *C. maculatus* and *P. interpunctella* over time, as determined using the OxySense 5250i instrument.

The average life cycle (from egg to adult emergence) was 22 days for *C. maculatus* and 37 days for *P. interpunctella* (Table 1). The average cumulative oxygen consumption obtained by the Oxysense instrument for *C. maculatus* (egg to adult stage) was 8.3 ml per insect. By comparison, the average cumulative consumption by *P. interpunctella* (26.9 ml/insect) was significantly higher than that of *C. maculatus* (Mann-Whitney *U* Test: Z = 6.34; p-value < 0.00001). The maximum rate of oxygen consumption for *C. maculatus* over a period of 8 days (day 9 through 16) was 0.8 ml/day, while *P. interpunctella* had a maximum rate of oxygen consumption of 1.4 ml/day over a period of 18 days (day 12 through 29) (Fig. 1 & Table 1). The results for maximum rate of oxygen consumption for the *C. maculatus* and *P. interpunctella* were not significantly different (Mann-Whitney *U* Test: Z = 1.75; p-value = 0.08012). The average daily rate of oxygen consumption for *C. maculatus* was 0.4 ml over a period of 22 days while that of *P. interpunctella* was 0.7 ml over a period of 37 days (Fig. 1 and Table 1). There was no significant difference between the average daily rate of oxygen consumption for *C. maculatus* and *P. interpunctella* (Mann-Whitney *U* Test: Z = 1.22; p-value = 0.22246).

**Table 1:** Average life cycle, average cumulative oxygen consumption, maximum rate of oxygen consumption, and average daily rate of oxygen consumption, by the *C. maculatus* and *P. interpunctella*. Means were compared using the Mann-Whitney *U* test. Means followed by the same letter in the same column are not significantly different (P < 0.05).

Insect	Avg. Life Cycle (days)	Avg. cumulative oxygen consumption (ml/insect)	Max. rate oxygen consumption (ml/day/insect)	Avg. daily rate of oxygen consumption (ml/day/insect)
Cowpea Bruchid (n = 22)	22	8.3 ± 0.3a	0.8 ± 0.1a	0.4 ± 0.2a
Indian Meal Moth (n = 36)	37	26.9 ± 0.7b	1.4 ± 0.3a	0.7 ± 0.3a
Mann-Whitney *U* Test		Z = 6.34; p < 0.00001	Z = 1.75; p = 0.08	Z = 1.22; p = 0.22

## 4 Discussion

In the present study, the OxySense 5250i allowed for the successful measurement of cumulative oxygen consumption by individual *C. maculatus*. Results were in good agreement with results reported using the Mocon PAC Check^®^ Model 325 Headspace analyzer (Murdock et al., 2012). No significant difference was observed in the data collected using the OxySense compared to the Mocon. With our success in corroborating Murdock et al.’s (2012) result together with our observations with *P. interpunctella*, we have demonstrated that the OxySense 5250i can be reliably used to measure the oxygen consumption of individual storage insect pests. The present study is the first to measure cumulative oxygen consumption by individual insects using the OxySense 5250i. Several other studies have used the OxySense to measure oxygen levels related to reproductive behavior of female *C. maculatus* and maize grain quality and aflatoxin accumulation under hermetic conditions (Tubbs et al., 2016; Yan et al., 2016). The OxySense has also been used in conjunction with acoustic studies of feeding behavior of *Sitophilus oryzae* (L.) (Njoroge et al., 2017). It is important to note that the trends observed in oxygen depletion using the OxySense under the same environment are not different from those observed using the Mocon (Martin et al., 2015; Williams et al., 2017).

It is not surprising that different insect species have different cumulative (lifetime) oxygen consumption requirements. As shown here, *P. interpunctella* requires much more oxygen to complete its development (26.9 ml vs. 8.3 ml) than does *C. maculatus*. This is expected as *P. interpunctella* has a larger body mass, a longer developmental cycle, and is an externally feeding pest that moves a lot as it grows and develops. To our knowledge, the majority of past studies have focused on short-term oxygen consumption (Emekci et al., 2002) and ours is among the first to look at lifetime cumulative oxygen consumption.

We observed similarities between the two insect species as it relates to the rate of oxygen consumption and the trend of oxygen consumption over time. The rate of oxygen consumption reaches the maximum on day 12 for *C. maculatus* and follows a logistic model. A study by Birch (1947) measuring the rate of oxygen consumption (mm^3^/insect/hour) of *Calandra oryzae* (L.) and *Rhyzopertha dominica* (Fab.) found that the rate of oxygen consumption rises as the larvae develop, reaching its maximum at the start of the pre-pupal stage, and finally dropping sharply during pupation. The time of the 2nd and 3rd instar (day 1–8) as well as the pupal stage (day 14–19) has been approximated for *C. maculatus* (Murdock et al., 2012). Our results also suggest that *C. maculatus* attains the maximum rate of oxygen consumption during the pre-pupal stage. The rate of oxygen consumption for *P. interpunctella* reached the maximum on day 26. Silhacek and Miller (1972) reported that *P. interpunctella* at 30 °C and 70% R.H. began the pupal stage around day 15, and emerged as adults on days 18–19. In our study, the life cycle of *P. interpunctella* was much longer, likely due to the lower temperature and R.H. of our experiments.

We found the average daily rate of oxygen consumption for *C. maculatus* to be 0.4 ml/day. *C. maculatus* began to show detectable oxygen use on day 9 (Fig. 1 and Table 1). Guedes et al. (2003) provided a similar model for the respiration rate (O2/larvae/day) of *C. maculatus* from day 0–7 as reported in this study (Fig. 1). The average daily rate of oxygen consumption for *P. interpunctella* was 0.7 ml/day. *P. interpunctella* began to exhibit detectable oxygen use on day 12 (Fig. 1 and Table 1). Thus *P. interpunctella* used oxygen at a rate nearly twice as fast as *C. maculatus*. Even so, the average daily rates of oxygen consumption for both insect species were not significantly different (Table 1). Ultimately, our findings on the average daily rate of oxygen consumption by *C. maculatus* and *P. interpunctella* confirm previously reported trends in insect oxygen consumption.

## Appendix A Supplementary data

Data profile Data profile
